# Addressing Safety, Quality, and Cost of Care Through a Telehealth Outpatient Transitional Care Model: Protocol for a Pragmatic Randomized Controlled Trial

**DOI:** 10.2196/71847

**Published:** 2025-09-05

**Authors:** Kate Davis, Sepehr Shakib, Greg Sharplin, Lachlan Darch, Nicholas Marlow, Marion Eckert

**Affiliations:** 1 Rosemary Bryant AO Research Centre Clinical and Health Sciences University of South Australia Adelaide Australia; 2 Department of Clinical Pharmacology Royal Adelaide Hospital Adelaide Australia; 3 School of Biomedicine Department of Clinical Pharmacology University of Adelaide Adelaide Australia; 4 College of Nursing adn Health Sciences Flinders University Adelaide Australia

**Keywords:** transitional care model, multimorbidity, primary care, secondary care, nurse led

## Abstract

**Background:**

People with multimorbidity have complex health care needs, resulting in high health service use, hospital readmission rates, and support needs. To prevent unnecessary hospital readmissions, effective coordination during the transition from hospital to primary care is essential; the transitional care model (TCM) is an effective approach to achieve this. This study will adapt the TCM, focusing on a nurse-led telehealth-based follow-up transition coordination service to enhance continuity between hospital and primary care, aiming to reduce unnecessary hospital readmissions and improve patient transitions.

**Objective:**

This study aims to assess the impact of a TCM on 3-month readmission rates in people with multimorbidity after discharge in an Australian context. Other objectives include evaluating the rate of re-presentation to hospital and overall length of hospital stay within 1, 6, and 12 months of discharge from the index admission; conducting a cost analysis of the transitional service model of care; evaluating the patient experience with the transition service; assessing patients’ symptom burden before and after transitional support service intervention; and evaluating patients’ quality of life, self-efficacy, and symptom management before and after intervention.

**Methods:**

The study design is a multicenter, pragmatic randomized controlled trial of patients with multimorbidity; therefore, real-world clinical practices, and operations will be the considerations within the research design elements. A mixed methods approach using quantitative and qualitative data collection methods will be used. The study setting incorporates 2 hospitals, initially commencing at the Queen Elizabeth Hospital (a 355-bed acute and subacute teaching hospital) and then at the Royal Adelaide Hospital (an 880-bed acute care teaching hospital), both located within the Central Adelaide Local Health Network, South Australia. We will include 3 to 6 medical units and wards. The intervention will focus on nurse-led transition assessment and care planning and telehealth transition coordination support for people with multimorbidity for 6 to 10 weeks following hospital discharge.

**Results:**

This project received ethics approval (17554) on June 29, 2023, and was registered with the Australian New Zealand Clinical Trials Registry on February 15, 2024 (12624000142538). The study commenced on July 1, 2023; data collection started in February 2024 and was completed on March 31, 2025. Finalized results are expected in March 2026.

**Conclusions:**

The Central Adelaide Local Health Network currently lacks a process to assess or manage readmission risks for people with multimorbidity, despite evidence linking transitional care to reduced rehospitalizations. Our feasibility study highlighted the effectiveness of a transition coordinator role in supporting patients’ return to home and community. Progressing this work, an adapted TCM, with telehealth-based follow-up and home and health care support, will enhance continuity between hospital and primary care, aiming to reduce unnecessary readmissions and improve patient transitions.

**Trial Registration:**

Australian New Zealand Clinical Trials Registry (ANZCTR) ACTRN12624000142538; https://www.anzctr.org.au/Trial/Registration/TrialReview.aspx?id=383721

**International Registered Report Identifier (IRRID):**

DERR1-10.2196/71847

## Introduction

### Background

Multimorbidity, the presence of 2 or more chronic conditions, is common and becomes more common with increasing age [1]. It is estimated that 8% of Australians (9.7 million people) had 2 or more chronic disease conditions in 2022. This ranged from 11% of people aged between 0 and 14 years to 79% of people aged ≥85 years [2]. Due to increasing life expectancy and improvements in health care, the prevalence of multimorbidity is rising [3,4]. People with multimorbidity have health outcomes characterized by functional decline, decreased quality of life, and increased mortality [5]. Multimorbidity is also costly for health systems and society due to associations with high patient readmission rates [1], high health care use [6,7], and decreased productivity [8-10]. Historically, the health system has been underpinned and strengthened by the primacy of the single disease model of illness [4,11]. However, there is consensus that the single disease model is unsuitable for people with multimorbidity; a coordinated model of transitional care at the primary and secondary health care interface is required to reduce health service use, hospital readmission, and care fragmentation and support positive patient experiences [4,12].

Several transition interventions have been trialed to reduce high short-term readmission rates [13,14]; however, no discrete intervention or bundle of interventions has been found to reliably reduce rehospitalization. Beneficial interventions for patients with multimorbidity include transition coaches, home visits, and postdischarge telephone calls. However, these patients also often take multiple medications (polypharmacy) and are frequently readmitted due to medication-related issues [15,16]. Depression and anxiety are also common in this group and can lead to poorer health outcomes [17,18]. To address these challenges, interventions such as home medication reviews and mental health care plans may improve overall care for patients with multimorbidity. Nonetheless, services required by people with multimorbidity go beyond their needs in relation to polypharmacy management. Services need to support continuity of care, including multifaceted, time-limited transitional care and multiple health and social service supports and coordination [19,20].

Many services that were previously only provided face-to-face or through outpatient services have successfully, after the COVID-19 pandemic, transitioned to a telehealth service [21]. Still, issues with telehealth delivery persist, including device and internet capabilities at the end of the consumer, the unfamiliarity of the user interface, and uncertainty regarding the cost-effectiveness at the end of the provider [21,22].

Although intensive interventions, including multidisciplinary care and home visits, have been shown to reduce short-term readmissions, they may not be cost-effective in all settings and are difficult to implement across an organization. Low-intensity interventions, such as phone calls, are easy to implement; however, they have not been shown to impact readmission rates by themselves [23]. What is needed is a low-cost intervention that combines multiple interventions into a coordinated package, which can be delivered without requiring face-to-face or outpatient consultations and which, if successful, can be readily spread across an organization.

The transitional care model (TCM) is a nurse-led multidisciplinary model of care developed at the University of Pennsylvania [24]. It focuses on improving the transition of older adults with complex health needs from hospital to home, aiming to reduce hospital readmissions, improve overall patient outcomes, and demonstrate lower overall health care costs by providing comprehensive coordinated care during the critical period following hospital discharge [19,25,26]. Through personalized care coordination, comprehensive assessments (including risk of readmission), ongoing time-limited follow-up, nursing care, and interventions, the TCM has proven effective in enhancing patient care quality while lowering health care costs [26].

To date, there is no evidence that the TCM has been implemented or adapted for implementation within the Australian health care system. However, related transitional care interventions have been trialed. An umbrella review by Berthelsen et al [27], which synthesized findings from 59 randomized controlled trials and 3 quasi-experimental studies relating to key components of the TCM, included 5 systematic reviews conducted in Australia. In these reviews, 5 Australian studies were identified. However, these studies were not direct implementations of the TCM.

The Australian studies included the following: (1) evaluations of general practitioner (GP) involvement in discharge planning to reduce emergency service use [28,29]; (2) a care coordination intervention not based on the TCM [30]; (3) several occupational therapy–focused interventions [31]; and (4) a study related to residential care management [32]. Berthelsen et al [27] also refer to broader evidence regarding GP engagement in transitional care, citing studies such as Allen et al [33], Manderson et al [34], Morkisch et al [19], and Parker et al [35].

In addition, Australia has implemented various care coordination programs, such as Planned Care for Better Health [36] and the inTouch Residential Aged Care Facility Pathway [37], although these initiatives are not based on the TCM. Other programs related to hospital discharge, such as those reported by Courtney et al [38], also diverge from the TCM framework.

A recent feasibility study examined a nurse-led transition care coordinator role designed to improve the quality of transitional care for individuals with multimorbidity at risk of hospital readmission [39]. The study was successful, providing evidence of the role’s acceptability, feasibility, fidelity, and sustainability from both patients and staff in the Australian context. Results highlighted a gap in local health service provision, whereby there was no current process for assessing or managing the risk of readmission for people with multimorbidity, despite international evidence linking effective transitional care to reduced rehospitalization rates [39]. In addition, the study showed that the participants demonstrated significant support needs during the transition period, suggesting that a crucial shift is needed from traditional discharge planning to more tailored transition planning and care coordination for people with multimorbidity [39].

Australia does not have the TCM embedded within its acute care services. This is significant because transition coordination between the primary and secondary health care sectors has been shown to decrease hospital readmissions and increase primary health care use [40]. This study is being implemented at 2 acute hospital sites, both located within the Central Adelaide Local Health Network (CALHN), South Australia; the purpose of the study is to implement an adapted nurse-led TCM to minimize unnecessary hospital readmissions and emergency department presentations for people with multimorbidity [12].

### Aim and Objectives

The aim of this study is to develop and test an adapted nurse-led transition model of care to optimally support people living with multimorbidity after hospital discharge via telehealth and ensure continuity of care between the secondary (acute) and primary health care sectors to minimize direct contact with hospital services.

The objectives of this study are as follows:

- To implement an adapted nurse-led TCM intervention
- To assess the intervention’s impact on the rate of re-presentation to the hospital (emergency department or hospital admission) within 3 months of discharge from the index admission
- To quantify the rate of re-presentation to hospital (emergency department or hospital admission) within 1, 6, and 12 months of discharge from the index admission
- To calculate a cost analysis of the transition service model of care
- To evaluate the participants’ symptom burden, self-efficacy, quality of life, and experience of continuity of care in relation to the intervention

### Hypothesis

We hypothesize that the provision of a nurse-led transition support service (after hospital discharge) for patients with multimorbidity will decrease the short-term hospital readmission rate for this patient cohort.

## Methods

### Ethical Considerations

This project received ethics approval on June 29, 2023, from the CALHN Human Research Ethics Committee (17554). It was registered with the Australian New Zealand Clinical Trials Registry on February 15, 2024 (12624000142538). Written informed consent will be obtained from all participants to ensure that they understand the study requirements, risks, and benefits. If potential participants accept an invitation to participate in the study, they will be provided with a participant information sheet and consent form for consideration. They will then be approached 24 hours later to confirm their willingness to participate. The study commenced on July 1, 2023; data collection started in February 2024 and was completed on March 31, 2025. Finalized results are expected in March 2026.

### Design and Setting

This study is a multicenter pragmatic randomized controlled trial (pRCT) of patients with multimorbidity for deployment at 2 acute hospital sites, initially commencing at The Queen Elizabeth Hospital (a 355-bed acute and subacute teaching hospital) and then at The Royal Adelaide Hospital (an 880-bed acute care teaching hospital), both located within the CALHN, South Australia. Between 3 and 6 medical units or wards will be included per site. This pRCT uses a mixed methods (quantitative and qualitative data collection) approach.

### Data Collection

The pRCT will use a mixed methods approach to data collection, primarily quantitative with a qualitative descriptive component. Data will be gathered through validated patient assessment and transition instruments (Table 1), electronic medical record reviews, phone call support and monitoring, and interviews.

**Table 1:** Data collection instruments, characteristics, and analysis methods.

Instrument	Number of items and response options	Analysis method
Community Assessment Risk Screen [41,42]	Three items (ranging 0-9): 0-3 (low risk); 4-9 (high risk)	Quantitative
Customized transition nursing assessment [43]	A total of 28 summary items	Quantitative and qualitative: open-ended narrative comments from participants
EQ-5D-3L health questionnaire [44]	Five dimensions: mobility, self-care, usual activities, pain or discomfort, and anxiety or depression and patient’s self-rated health on a vertical visual analog scale	Quantitative and qualitative: open-ended narrative comments from participants
Patient Continuity of Care Questionnaire (short) [45]	A total of 12 items	Quantitative and qualitative: open-ended narrative comments from participants
Barthel Index [46]	A scale describing 10 tasks scored according to the amount of time or assistance required by the patient; total score from 0 to 100, with lower scores representing greater nursing dependency	Quantitative
Edmonton Frailty Scale [47,48]	A scale sampling 10 domains, where the maximum score is 17, representing the highest level of frailty	Quantitative
Kessler Psychological Distress Scale [49]	A 10-Item measure designed to assess nonspecific psychological distress	Quantitative
Malnutrition Universal Screening Tool [50]	Three independent criteria are assessed: BMI score, weight loss score, and acute disease effect score; all scores are added, where 0 indicates low risk, 1 indicates medium risk, and a score of 2≥ indicates high risk	Quantitative
General Self-Efficacy Scale [51]	A 10-item scale for a self-rated measurement of self-efficacy; total score between 10 and 40, with higher scores indicating higher self-efficacy	Quantitative and qualitative: open-ended narrative comments from participants
Edmonton Symptom Assessment System [52]	Measures symptom burden of chronic disease and has evolved into an 11-point numeric rating scale, ranging from 0 (no symptom) to 10 (worst possible)	Quantitative

As no suitable preexisting tools were available, customized instruments were developed in REDCap (Research Electronic Data Capture; Vanderbilt University) to capture transition assessment data, medical record readmission data, and phone call monitoring data [53]. Table 1 provides an overview of the validated data collection instruments, their key characteristics, and the planned analysis. All 10 instruments will collect quantitative data, with 4 also incorporating qualitative data.

Throughout the study, researchers and Supporting Transitions and Referrals nurses (STARnurses; recruited to support transitions and referrals) will prioritize patient sensitivity, build rapport, and minimize participant burden.

### Qualitative Data Analysis

Thematic analysis, following the methodology by Braun and Clarke [54], will be used to categorize, analyze, and synthesize data from interviews and qualitative survey responses. Up to 15 interviews will be conducted or until thematic saturation is achieved.

Interview tools, including 12 open-ended questions, will be designed, piloted, and validated by us before data collection. All interviews will be audio recorded and transcribed using artificial intelligence transcription software, with transcriptions reviewed and validated by participants. Once finalized, transcripts will be uploaded to NVivo (Lumivero) software for coding and analysis. To protect confidentiality, all personal and identifiable information will be removed, and participants will be assigned pseudonyms (numerical IDs).

Each transcript will be systematically reviewed and coded, followed by an inductive categorization process. To support theme development, transcripts and categorized data will be read multiple times, allowing for the identification of emerging themes within the categories.

### Statistical Analysis

An intention-to-treat approach will be used. Linear regression analyses will be conducted to estimate the effects of the intervention on primary and secondary outcomes. A fixed effects model will be used to account for stratification at the hospital and unit levels. Sensitivity analyses will be performed to assess the robustness of the findings. Table 2 presents the alignment of data analysis with primary and secondary study outcomes.

**Table 2:** Primary and secondary outcomes—measurement and statistical analysis.

Outcome					Data source, measurement, and analysis
**Primary**					
	The percentage of patients having hospital re-presentation (emergency department presentation with or without hospital admission) within 3 months of discharge from the index admission			Re-presentation data available from the electronic medical record Statistical analysis: comparison of proportions between treatment and control groups and time to re-presentation analysis using the log-rank test with the method of Kaplan-Meier [55]	
**Secondary**					
	**Health care use**				
		The rate of hospital re-presentation within 1, 6, and 12 months of discharge from the index admission	Readmission data available from electronic medical record Rate defined as the number of re-presentations over a unit of time (eg, 1 y) Statistical analysis: nonparametric comparison of rates of re-presentation between treatment and control groups		
		Overall length of hospital stay within 1, 3, 6, and 12 months of discharge from the index admission	Readmission data available from electronic medical record Defined as the cumulative number of days spent in hospital over a unit of time (eg, d/y) Statistical analysis: nonparametric comparison of rates of re-presentation between treatment and control groups		
		Cost of transition service	Economic benefits of the intervention will be confirmed through a comparison of subsequent rehospitalization costs in the treatment and control groups		
	**Model of care and patient outcomes**				
		Patient-reported symptom burden	ESAS^a^ [52] (nonparametric comparison of responses between treatment and control groups)		
		Patient symptom management and self-efficacy	ESAS [52] GSE^b^ [51] (nonparametric comparison of responses between treatment and control groups)		
		Quality of life	EQ-5D-3L health questionnaire [44]		
		Patient experience, satisfaction, and perceived continuity of care	PCCQ^c^ (short) [45] (nonparametric comparison of responses between treatment and control groups)		
	**Organization of care**				
		Implementation of components of the TCM^d^	Economic benefits: cost calculations of comparison between control and intervention hospital readmissions from the index admission Includes TCM intervention cost compared with usual care cost		
		Implementation of the transition action plan for all patients	Customized transition action plan developed		

^a^ESAS: Edmonton Symptom Assessment System.

^b^GSE: General Self-Efficacy Scale.

^c^PCCQ: Patient Continuity of Care Questionnaire.

^d^TCM: transitional care model.

### Sample Size Estimation and Justification

We anticipate that the enrollment of approximately 200 patients over 15 months will be feasible and provide a sufficient sample size to achieve the primary outcome, with the anticipated baseline readmission rate in the control group and absolute reduction in the intervention group (Table 3). Enrollment commenced in February 2024 and was completed on March 31, 2025. Given that the primary end point will be derived from health information systems in South Australia and that the study has an intention-to-treat approach, there will be a need to consider a larger sample size, taking into account withdrawals, dropouts, or loss to follow-ups.

**Table 3:** Sample size per group by baseline readmission rate and absolute reduction in readmission with 80% power (α=.05).

Absolute reduction in readmission rate in the intervention group (%)	Baseline readmission rate in the control group at 3 mo, n		
	35%	40%	45%
15	136	150	160
20	70	79	86
25	40	47	52

### Randomization

The randomization will occur at the individual level. The participants will be randomized 1:1, with stratification at the ward or unit level to intervention or control, using REDCap, a secure, web-based software platform designed to support data capture for research studies [53].

### Control

Participants in the control group will receive usual care, which is defined as the “usual follow-up services” planned while the patient is in hospital, before discharge. Transition care is not implemented after discharge from the hospital (usual care). Usual services are planned or initiated while the participant is in the hospital in preparation for discharge, but provision of these services is not followed up by the STARnurses.

### Eligibility Criteria

The eligible population includes individuals aged ≥18 years with multimorbidity who fulfill all inclusion criteria and do not meet any of the exclusion criteria. For the purposes of the inclusion criteria, multimorbidity is defined as the coexistence of 2 or more chronic conditions [56] (Textbox 1).

**Inclusion and exclusion criteria.**

**Inclusion criteria**

- Inpatients (ward or emergency department)
- Sufficient cognitive function and English language skills to provide informed consent and complete assessments
- Ability to engage in telehealth
- A history of either diabetes with cardiovascular disease or comorbidity in minimum 3 of the following illness domains:
  Diabetes—type 1 or type 2
  Cardiovascular disease—symptomatic atherosclerotic disease (ischemic heart disease, cerebrovascular disease, peripheral vascular disease, symptomatic valvular heart disease, or atrial fibrillation)
  Chronic cardiac failure
  Psychiatric illness, including mood or anxiety disorders
  Respiratory disease, including chronic obstructive pulmonary disease, asthma, or interstitial lung disease
  Kidney disease resulting in chronic renal impairment, with creatinine clearance ≤30 mL/min
  Current malignant neoplasm

**Exclusion criteria**

- Patients living in or likely to be discharged to a high-level residential care facility for older adults
- Patients enrolled in a comprehensive management program upon discharge, for example, a formal rehabilitation program, rehabilitation in the home, heart failure outreach service, chronic obstructive pulmonary disease pulmonary rehabilitation, hospital in the home, or receiving disability or community psychiatric services, including care coordination
- Patients followed up through other local health network services providing community outreach, such as patients who are homeless
- Patients with a current history of illicit drug or alcohol dependence, which may interfere with their ability to engage with the program
- Patients with palliative intent and likely to have a life expectancy of <6 mo
- Patients due for elective readmission within 2 wk of current hospital discharge
- Insufficient cognitive function or English language skills to provide informed consent and complete assessments
- Patients who are found to have been in hospital for >3 wk during the index admission and are not transitional patients

### Identification and Recruitment of Participants

Several procedures to identify and recruit participants for the trial intervention have been incorporated into the study design.

Nursing, medical, pharmacy, and allied health leads and managers; ward staff; and patient flow and bed management meetings will be attended by the research leads and STARnurses to brief staff and explain all aspects of the research and introduce the STARnurses and their role. Emails and flyers will be sent by the research leads to medical, nursing, pharmacy, and allied health leads as well as relevant clinical staff.

Study criteria will be entered into the local electronic medical record to produce a trial list of potential participants. The STARnurses will review the list daily and follow up with relevant treating clinicians. Daily rounds will be conducted at trial wards, and nursing staff will liaise with medical staff to identify patients. Huddles will be attended, and nursing staff will liaise with medical staff to identify patients.

The patient’s clinical team will be consulted first, and if permission is granted, a research team member will then approach the patient. They will be invited to participate in the study, and if they accept, they will be provided with a participant information sheet and consent form for consideration. The patient will then be approached 24 hours later to confirm their willingness to participate. Individual randomization using REDCap will take place following informed written consent [53].

It is acknowledged that participants who provide consent will have multimorbidity and may even be frail, and the collaboration with their multidisciplinary team will be an integral component of the intervention. Therefore, balancing the participant’s agency and self-efficacy and capability with their anxiety and hesitancy will require ongoing discussion between the participant and team members, along with their support.

### Intervention

The intervention involves establishing a transition coordination service led by nursing transition coordinators, who will support transitions and referrals, known as STARnurses. The intervention consists of 4 key phases.

The first phase involves in-hospital screening in which “risk of readmission” screening is conducted during the hospital stay, using the Community Assessment Risk Screen tool [41,42]. Patients who meet the inclusion criteria are invited to participate in the study.

The second phase involves in-hospital baseline assessment, transition planning, and participant goal setting (following participant research and information discussion, acceptance to participate, and consent procedure complete). A detailed inpatient baseline and transition assessment is performed to collaboratively develop personalized goals and a transition action plan. Researchers will use the following tools to assess participants’ transition needs: customized transition nursing assessment [43], Barthel Index [46], Edmonton Frailty Scale [47,48], Kessler Psychological Distress Scale [49], Malnutrition Universal Screening Tool scale [50], and General Self-Efficacy Scale [51]. The participant’s GP or practice nurse will be contacted and receive the transition action plan before the participant’s hospital discharge.

**Figure 1:**
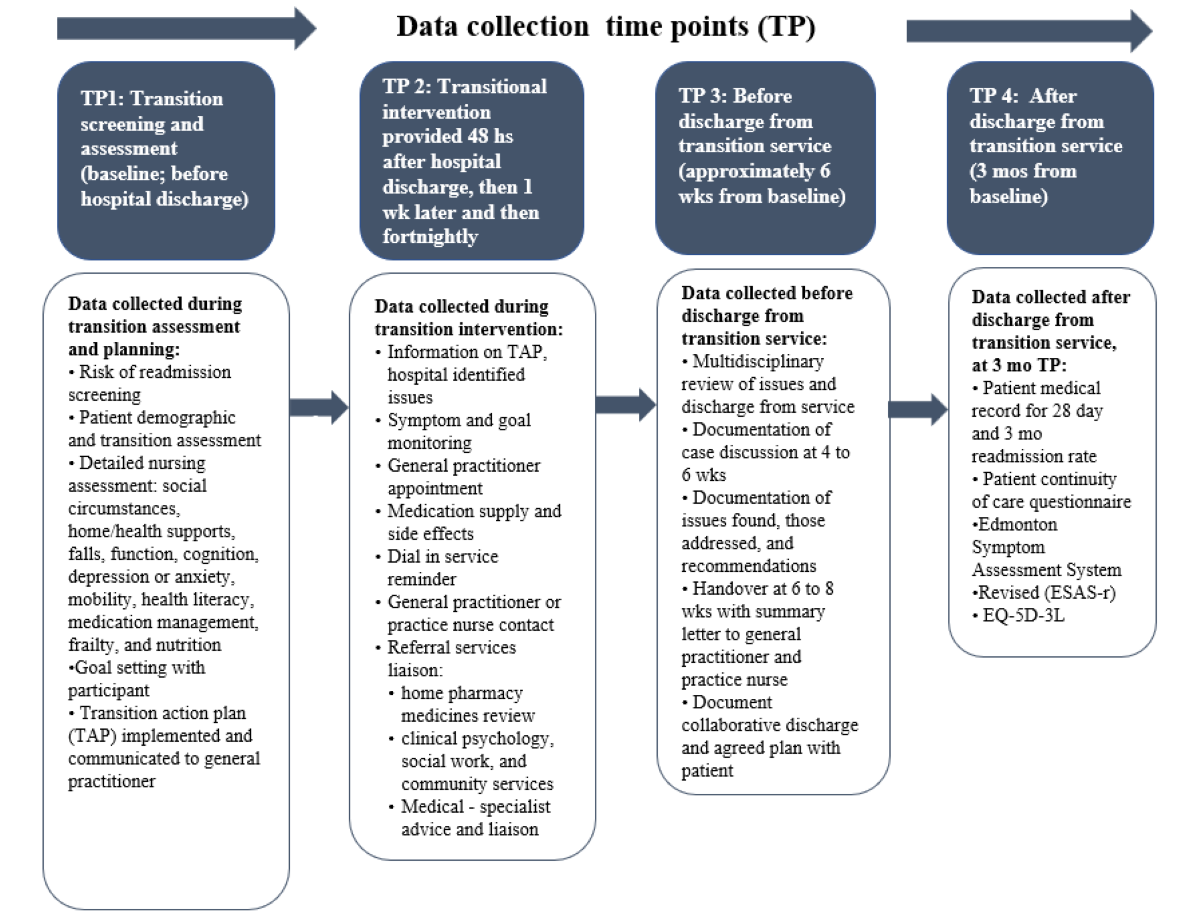
Interventions aligned with time points (TPs) of data collection and phone calls. ESAS-r: Edmonton Symptom Assessment System–Revised; TAP: transition action plan.

The third phase is posthospital discharge transition coordination. Participants will receive transitional care coordination, with phone calls or telehealth follow-up in the first 48 hours following discharge and then weekly and fortnightly (Figure 1).

The fourth phase involves handover to primary care. A multidisciplinary case discussion will occur approximately 4 weeks after discharge in preparation for handover to the GP or practice nurse and other specialist providers. The STARnurse will have a final consultation with the patient after the multidisciplinary case discussion (6-10 wk after discharge) to inform the patient of any outcomes or actions. Handover and written communication are provided to the GP or practice nurse to ensure the participant is stable in the community, and if not stable, follow-up care is required.

In this study, a key adaptation of the TCM is replacing the advanced practice registered nurse home visits with STARnurse telehealth or telephone follow-up. This follow-up is provided according to the intervention protocol (Figure 1). The feasibility of the STARnurse and transition coordinator role and the associated transition service was previously evaluated in an earlier feasibility study [24].

## Results

This project received ethics approval (17554) on June 29, 2023, and it was registered with the Australian New Zealand Clinical Trials Registry on February 15, 2024 (12624000142538). This study commenced on July 1, 2023, with data collection commencing in February 2024; data collection was completed on March 31, 2025. Finalized results are expected in March 2026.

## Discussion

### Anticipated Findings

The primary outcome of this research is decreasing the rate of hospital readmission within 3 months of discharge from the index admission in people with multimorbidity. In a prospective, quasi-experimental TCM study (N=172), improvements in all health status and quality of life measures were observed after intervention compared to before the intervention, and a significant decrease in total number of readmissions (TCM: 45 readmissions vs control: 60 readmissions; *P*<.041) and total hospital days (TCM: 252 d vs control: 351 d; *P*<.032) were observed at 3 months [57].

The secondary outcomes include the cost analysis of the transition service model of care. In a previous study, cost savings were demonstrated in relation to appropriate implementation of the TCM, whereby it was associated with a short-term decrease of US $439 per patient per month in total health care costs at 3 months and cumulative per-patient savings of US $2170 at 1 year (*P*<.037). In a cost comparison study in which the post–acute care costs of 3 care management interventions for hospitalized older adults with cognitive impairment and their family caregivers were compared (N202), the TCM had significantly lower costs than 2 other comparable care management interventions and has been associated with an estimated net savings of US $2972 per individual at 6 months [26].

People with multimorbidity are readmitted to the hospital more, spend more time seeking health care, and often receive fragmented care. When they are discharged from the hospital, they are often readmitted due to poor health care team communication, uncoordinated follow-ups, and not knowing whom to contact when they have symptoms. Supporting these patients as they transition to the community after hospital discharge with an adapted nurse-led TCM intervention will assist them to resume their normal lives more quickly and prevent unnecessary hospital contact.

This study is informed by TCM evidence and an earlier feasibility study [12,39]. Consequently, it represents an additional and focused investigation into a discrete and known area of health service provision. This focus has allowed researchers to refine the intervention to a practical and effective modality. The method responds to previously encountered challenges and locates the participant and their health care team at its center. It is anticipated that patients’ awareness of this approach will encourage their health care agency in a goal-directed and beneficial manner. The previous research was at a single large acute hospital; the sample size was small; and the design omitted the use of comparator groups. These parameters have been addressed in this study, enabling future generalizability of the findings.

### Limitations

Although the power of the research has been addressed through an appropriate cohort number, the 2 hospitals included in this study remain within the same local health network, which could emerge as a limitation later. At this point, an adapted nurse-led TCM is being trialed, posing infidelity to the original model; in addition, people with cognitive impairment have been excluded. However, this research is considered part of an overall plan to eventually embed the transition care model as standard care within the health system; consequently, each research project builds on demonstrated evidence.

Health sectors use diverse electronic record and patient data systems, which can lead to challenges with access and consistency. To address these challenges, this study is a pragmatic one, emphasizing relationship building [46] and fostering regular, consistent communication among the multidisciplinary team, the participants and significant others, and the health sectors as integral components of the nursing interventions.

### Conclusions

In this research, the role of the STARnurses is key, which spans the hospital, multidisciplinary teams, and the primary health care setting. Their collaborative role enables patient-centered care that is tailored to participants’ transition goals; it needs to empower them in the management of their own care. Evidence is clear that the TCM has a positive impact on reducing readmission rates through its enhanced care coordination and the management of patients in transition from the acute to the primary health care sector. However, the South Australian local health networks currently lack a structured process to evaluate the risk of hospital readmission at the time of discharge, even though readmissions among patients with multimorbidity are both costly and potentially avoidable. This research offers the opportunity to enhance continuity of care, minimize unnecessary rehospitalizations, significantly decrease overall health care use, and improve quality of life for people with multimorbidity. It also represents a significant opportunity to transform health care delivery, emphasizing a shift from discharge planning to an integrated model of transition planning and care.

## Abbreviations

CALHN: Central Adelaide Local Health Network
GP: general practitioner
pRCT: pragmatic randomized controlled trial
STARnurse: Supporting Transitions and Referrals nurse
TCM: transitional care model

## Data Availability

The datasets generated or analyzed during this study will be available from the corresponding author on reasonable request.
